# Differences in Immunohistochemical and Ultrastructural Features between Podocytes and Parietal Epithelial Cells (PECs) Are Observed in Developing, Healthy Postnatal, and Pathologically Changed Human Kidneys

**DOI:** 10.3390/ijms23147501

**Published:** 2022-07-06

**Authors:** Marin Ogorevc, Ivona Kosovic, Natalija Filipovic, Ivana Bocina, Marija Juric, Benjamin Benzon, Snjezana Mardesic, Katarina Vukojevic, Marijan Saraga, Boris Kablar, Mirna Saraga-Babic

**Affiliations:** 1 Department of Anatomy, Histology and Embryology, School of Medicine, University of Split, 21000 Split, Croatia; marin.ogorevc2@gmail.com (M.O.); ivona.kosovic@gmail.com (I.K.); natalija.filipovic@mefst.hr (N.F.); maarjur@gmail.com (M.J.); benjamin.benzon@mefst.hr (B.B.); smbrakus@gmail.com (S.M.); kvukojev@gmail.com (K.V.); 2 Department of Biology, Faculty of Science, University of Split, 21000 Split, Croatia; bocina@pmfst.hr; 3 Department of Paediatrics, University Hospital in Split, 21000 Split, Croatia; msaraga@kbsplit.hr; 4 Department of Medical Neuroscience, Faculty of Medicine, Dalhousie University, Halifax, NS B3H 4R2, Canada; boris.kablar@dal.ca

**Keywords:** podocytes, PECs, developing kidneys, WNT4, Notch2, Snail, CNF, FSGS

## Abstract

During human kidney development, cells of the proximal nephron gradually differentiate into podocytes and parietal epithelial cells (PECs). Podocytes are terminally differentiated cells that play a key role in both normal and pathological kidney function. Therefore, the potential of podocytes to regenerate or be replaced by other cell populations (PECs) is of great interest for the possible treatment of kidney diseases. In the present study, we analyzed the proliferation and differentiation capabilities of podocytes and PECs, changes in the expression pattern of nestin, and several early proteins including WNT4, Notch2, and Snail, as well as Ki-67, in tissues of developing, postnatal, and pathologically changed human kidneys by using immunohistochemistry and electron microscopy. Developing PECs showed a higher proliferation rate than podocytes, whereas nestin expression characterized only podocytes and pathologically changed kidneys. In the developing kidneys, WNT4 and Notch2 expression increased moderately in podocytes and strongly in PECs, whereas Snail increased only in PECs in the later fetal period. During human kidney development, WNT4, Notch2, and Snail are involved in early nephrogenesis control. In kidneys affected by congenital nephrotic syndrome of the Finnish type (CNF) and focal segmental glomerulosclerosis (FSGS), WNT4 decreased in both cell populations, whereas Notch2 decreased in FSGS. In contrast, Snail increased both in CNF and FSGS, whereas Notch2 increased only in CNF. Electron microscopy revealed cytoplasmic processes spanning the urinary space between the podocytes and PECs in developing and healthy postnatal kidneys, whereas the CNF and FSGS kidneys were characterized by numerous cellular bridges containing cells with strong expression of nestin and all analyzed proteins. Our results indicate that the mechanisms of gene control in nephrogenesis are reactivated under pathological conditions. These mechanisms could have a role in restoring glomerular integrity by potentially inducing the regeneration of podocytes from PECs.

## 1. Introduction

The metanephric mesenchyme (MM) is the source of the nephrons in the definitive human kidney. After induction, the cells of the MM condense and undergo a mesenchymal-to-epithelial transition (MET), which results in the formation of renal vesicles. These vesicles grow and further differentiate, passing through several morphological stages (comma-shaped body, S-shaped body, capillary-loop stage, etc.) until they finally give rise to mature nephrons [1,2]. The proximal end of the developing nephrons differentiates into podocytes and parietal epithelial cells (PECs), which form the Bowman’s capsule [3].

Podocytes are interesting cells, showing both epithelial and mesenchymal characteristics at the same time. They can be first discerned at the S-shaped stage when, following MET, they acquire epithelial features [4]. However, already during the capillary loop stage, they undergo the reverse process of epithelial-to-mesenchymal transition (EMT) and regain mesenchymal characteristics, while losing some but not all epithelial markers [5]. However, of the three known types of EMT, none can fully explain the final outcome of podocyte development [6]. Another feature that distinguishes podocytes from all the other nephron and glomerular cells is their inability to divide, as they are terminally differentiated cells arrested in a postmitotic state. In fact, the forced re-entry of podocytes into the cell cycle results in cell death due to mitotic catastrophe [7]. The loss of podocytes seems to be a key factor in glomerular disorders [8] and the progression of chronic kidney disease (CKD) leading to end-stage kidney disease [1]. It was, however, demonstrated that lost podocytes could be replaced by newly formed ones, which suggested that the source of these new podocytes is the PECs [9], which can be first distinguished in the S-shaped body as they begin expressing different markers [10]. They form a simple squamous epithelium, which is continuous with the proximal convoluted tubule at the urinary (tubular) glomerular pole [11]. Near the vascular pole, some cells of the parietal layer express markers and have ultrastructural features of podocytes, comprising about three-quarters of human glomeruli [12]. Although PEC pathology is mostly associated with crescentic glomerulonephritis, both the proliferation and detachment of PECs were described in other glomerular diseases such as focal segmental glomerulosclerosis (FSGS) [13,14,15]. Cells expressing stem cell markers near the urinary pole of Bowman’s capsule could differentiate into podocytes under appropriate conditions [16]. Lineage tracing studies have also shown that PECs could be the source of podocyte progenitors both during normal nephrogenesis [17] and under pathological conditions [18]. It was suggested that podocyte progenitors migrate along with the parietal layer of the Bowman’s capsule towards the vascular pole, where they can cross onto the glomerular tuft and reach their final destination [17]. However, this process was shown to be functionally inefficient. Ultrastructural studies disclosed cellular processes spanning the urinary space and connecting the glomerular tuft with the wall of Bowman’s capsule. There are controversies about whether those processes originate from podocytes [19,20] or PECs [21] or whether they belong to differentiating podocyte progenitors [22]. By now, the formation of these cellular bridges has been demonstrated in rat models of nephritis [23] suggesting that they may represent additional pathways for the replacement of damaged podocytes [18]. However, there is no evidence of the existence of these bridges during normal human nephrogenesis.

There are several markers of podocytes and/or PECs that were shown to change during normal nephrogenesis and glomerulogenesis, as well as under pathological conditions. One of them is nestin, an intermediate filament protein expressed in different precursors and stem cells during early development [24]. During earlier stages of development, nestin is expressed in both podocytes and PECs, but in the later stages and in postnatal kidneys, its expression is present only in podocytes [25]. Nestin expression is more intense in podocytes of kidneys affected by congenital nephrotic syndrome of the Finnish type (CNF) compared to podocytes of healthy kidneys. However, the percentage of nestin-positive podocytes does not differ between healthy and CNF kidneys [25,26].

Notch receptors are highly conserved transmembrane proteins involved in direct cell–cell signaling that affect proliferation, apoptosis, and differentiation during the development of a multitude of organs [27,28]. Notch2 is normally expressed in developing nephrons but its expression significantly decreases in mature kidneys [29]. An increased expression of Notch2 has been described in the podocytes and PECs of both mice and humans with nephrotic glomerulopathies, indicating its possible role in the pathogenesis of these diseases [30,31,32,33].

Wnts are cysteine-rich paracrine factors involved in many developmental processes such as axis formation [34], organogenesis [35], and the proliferation of stem cells [36]. Wnt4 is the main nephrogenesis control signal secreted by the metanephric mesenchyme as it begins to undergo MET [37,38]. Changes in the expression of Wnt4 signaling have been described both in experimental models and human samples of CKD [39,40].

Snail is a member of a zinc-finger transcription factor superfamily necessary for the formation of the mesoderm and neural crest [41], EMT [42], left–right asymmetry [43], and cell survival and division [44]. Snail expression is present in mouse metanephric mesenchyme; however, it becomes downregulated prior to MET and is not present in the healthy mature kidney tissue [45]. Upregulation of Snail expression has been shown in podocytes of the nephrotic kidneys of mice, rats, and humans, implying its potential role in the dedifferentiation and pathological transformation of podocytes [6,46,47].

The summarized information about the investigated proteins can be found in Table 1. Information about the differentiation of podocytes and PECs during human nephrogenesis is limited since most investigations have been performed on animal models or pathologically changed kidneys. Our study was undertaken in order to analyze changes in the expression of different markers in podocytes and PECs during successive stages of human kidney development, as well as in healthy postnatal and nephrotic kidneys. These specific markers were chosen as they have an important role in the process of EMT, which is crucial for podocyte differentiation. We also investigated the presence of intercellular communications between the podocytes and PECs during normal development and in kidneys affected by CNF and FSGS.

## 2. Results

The first immature glomeruli consist of several cell populations including podocytes, and the mesangial cells and endothelial cells of the glomerular tuft capillaries. Podocytes are continuous with PECs at the vascular glomerular pole. PECs mostly consist of simple squamous epithelium, whereas some of them show a cuboidal appearance, particularly those situated towards the vascular pole (also called peripolar cells). In places, cellular extensions bridge the urinary space between the podocytes and PECs (schematic drawing, Figure S1). Lower magnification images demonstrating the tissue architecture of the analyzed samples can be found in the Supplementary Materials (Figure S2).

### 2.1. Semi-Thin Sections and Electron Microscopy of Developing, Postnatal, and CNF Human Kidneys

**Figure 1:**
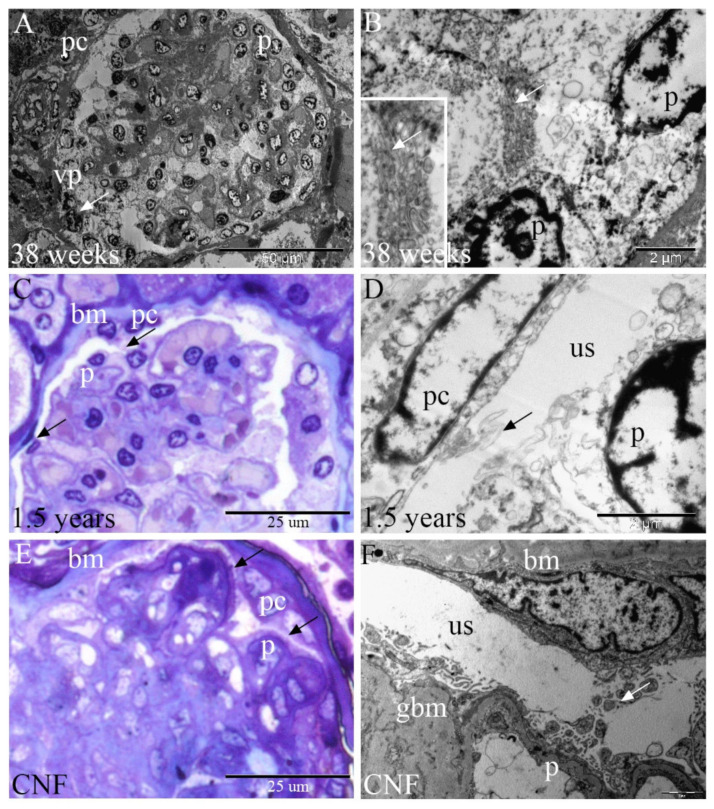
Semi-thin and ultrastructural sections show ultrastructural characteristics of late fetal, postnatal, and CNF kidneys. podocytes (p), PECs (pc), vascular pole (vp), basement membrane (bm), urinary space (us), and glomerular basement membrane (gbm). (**A**) 38th developmental week; glomerular tuft podocytes and PECs widely communicate (arrow) at the vascular pole. (**B**) 38th developmental week; numerous interdigitating cytoplasmic processes (inset, arrows) connect adjacent podocytes. (**C**) Postnatal healthy kidneys; podocytes communicate by cellular bridges (arrows) with PECs. (**D**) Postnatal healthy kidneys; long cytoplasmic extensions of podocytes and PECs bridge (arrow) the urinary space. (**E**) CNF kidneys; podocytes and PECs are connected by cytoplasmic extensions or by closely apposed cellular areas (arrows). (**F**) CNF kidneys; podocytes send numerous thick cytoplasmic extensions (arrow) through the urinary space towards the PECs.

In the 38th week of kidney development, low-magnification electron micrographs show the whole glomerulus widely connected to the vascular pole. Numerous cytoplasmic extensions between the podocytes and PECs bridge the rest of the urinary space (Figure 1A).

A higher magnification of the podocytes in the 38th developmental week shows numerous interdigitating cytoplasmic processes between the neighboring glomerular podocytes. (Figure 1B).

Semi-thin sections of healthy postnatal human kidneys show a centrally positioned glomerulus containing blood vessels, mesangial cells and podocytes, and PECs of Bowman’s capsule lying on the basement membrane. PECs are either in the form of simple squamous epithelium or cuboidal cells, which bridge the urinary space by cytoplasmic extensions, connecting them to the podocytes (Figure 1C).

In higher magnification electron micrographs of the postnatal kidneys, cytoplasmic extensions are observed between the PEC population and podocytes on the surface of the glomerulus. It appears that the interdigitating cytoplasmic protrusions from both types of cells join in the urinary space (Figure 1D).

In the kidneys affected by CNF, semi-thin sections show longer areas of association between the podocytes and PECs and a thickened basement membrane. Some PECs are cuboidal or even cylindric in shape. An accumulation of the extracellular matrix is observed in the glomerulus (Figure 1E).

In electron micrographs of CNF kidneys, PECs contact podocytes using the abundant thick cytoplasmic extensions that bridge the urinary space. A thickening of the basement membrane and extracellular matrix accumulation are also observed (Figure 1F).

The average length of intercellular bridges in x40 magnification immunofluorescent microphotographs was 24.9 ± 10.486 µm (SD). The smallest bridge measured 9.95 µm, whereas the largest was 44.32 µm. Analysis of high-magnification TEM images revealed even smaller bridges, the smallest measuring 1.878 µm in length and 0.821 µm in width at its narrowest point.

### 2.2. Proliferation of Glomerular Cells (Ki-67 Staining) in Developing, Postnatal, and Human Kidneys Affected by CNF and FSGS

**Figure 2:**
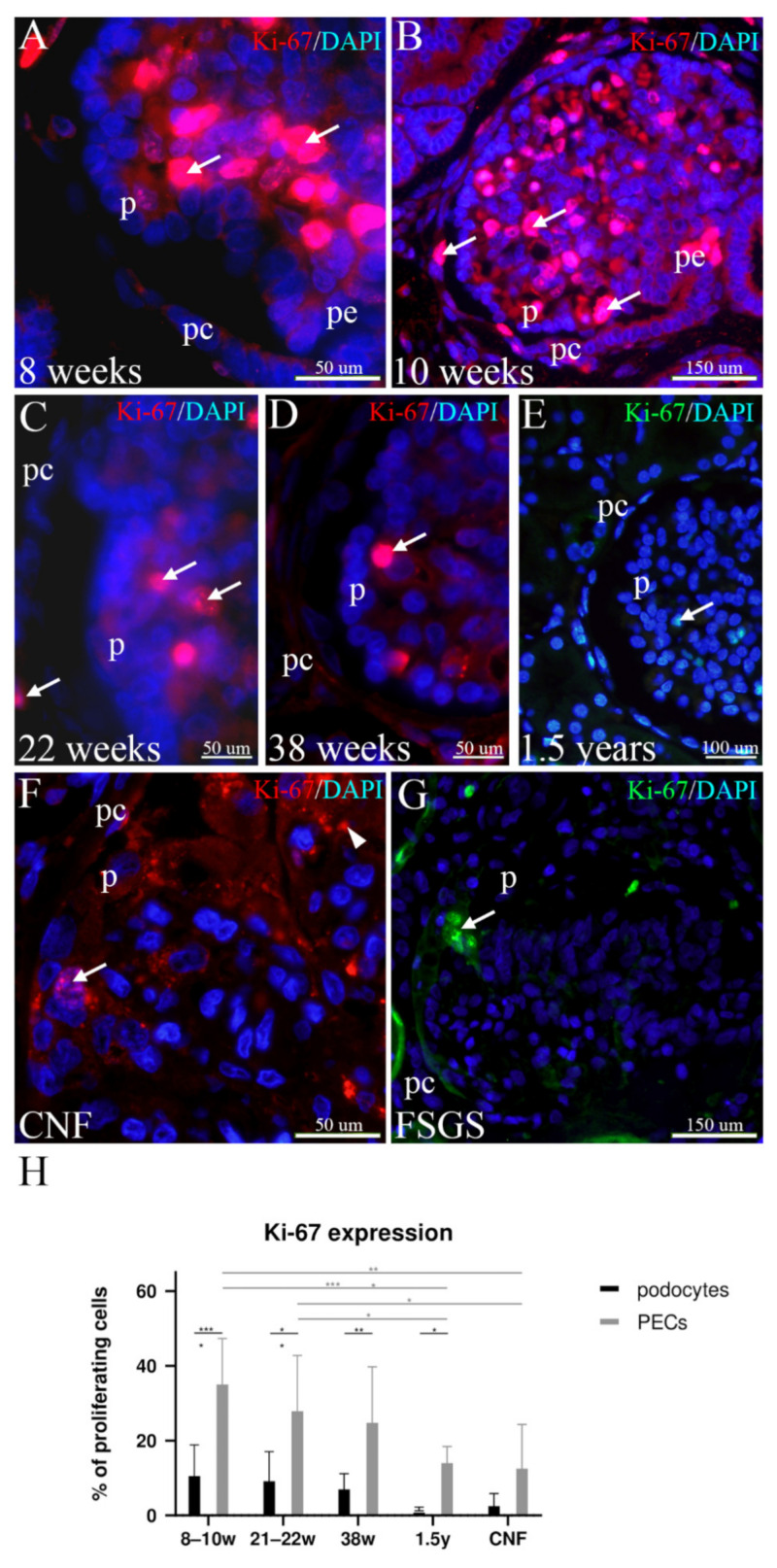
Proliferating (Ki-67-positive) podocytes and PECs during human kidney development, in postnatal, healthy, and pathologically changed kidneys (CNF, FSGS); podocytes (p), PECs (pc), peripolar cells (pe). (**A**–**D**) Normal developing kidneys; Ki-67-positive cells (arrows) predominate in the glomerular bulk in the earlier stages and characterize PECs, podocytes, and peripolar cells. (**E**) Postnatal healthy kidneys; a reduced number of Ki-67-positive cells (arrow) is observed in the podocytes and PECs. (**F**) CNF kidneys; proliferating cells are seen only occasionally (arrow) and an accumulation of unspecific staining can be observed in disintegrating cells (arrowhead). (**G**) FSGS kidneys; groups of proliferating cells (arrow) connect PECs and podocytes. (**H**) Graph showing percentages of Ki-67-positive cells (podocytes and PECs) during development, postnatally, and in CNF and FSGS samples. Error bars show SD. * *p* < 0.05; ** *p* < 0.01; *** *p* < 0.001.

In the 8th week of kidney development, the first immature glomeruli develop in the central part of the kidney tissue. Although the central part of the glomerulus (glomerular bulk) shows numerous proliferating cells, the surface layer of cuboidal podocyte precursors shows proliferation only occasionally. At the same time, PECs contain several proliferating cells, particularly in the transitional area between the podocytes and PECs (peripolar region). Some PECs have an oval appearance and bridge the developing urinary space, thus connecting them with the podocytes on the surface of the glomerular tuft (Figure 2A).

By the 10th developmental week, the glomeruli become larger and the number of proliferating cells increases primarily in the glomerular bulk (mesangial and endothelial cells) but not in the podocyte cell population. In contrast, PECs show strong proliferating activity, particularly in the peripolar region. In several places, the close association between the proliferating or non-proliferating PECs and glomerular podocytes can be observed to bridge the urinary space (Figure 2B).

During further fetal development, proliferation decreases both in the glomerular bulk and in the PECs (Figure 2C), whereas by the end of the fetal period, PECs proliferate only occasionally (Figure 2D). In the postnatal period, proliferating cells are seen in the bulk of the glomerulus, however, they are missing in the surface cells corresponding to podocytes. Proliferating PECs are rarely observed (Figure 2E).

In CNF kidneys, proliferating mesangial cells in the bulk of the glomeruli are seen only occasionally, whereas neither podocytes nor PECs show increased proliferations (Figure 2F).

In contrast to CNF, kidneys affected by FSGS show groups of proliferating PECs bridging the urinary space in the affected glomeruli. In addition, individual proliferating cells are observed inside the glomerular tuft (Figure 2G).

Statistical analysis reveals the strongest proliferation of podocytes (10%) and particularly PECs (35%) in the earliest developmental stages. During further development, the proliferation of both podocytes and PECs gradually decreases to reach only 1% of proliferating podocytes and 18% of PECs in the postnatal period. Throughout the investigated period, the proliferation of PECs is significantly higher than the podocyte proliferation. In CNF kidneys, the number of podocytes entering the cell cycle is higher (3%) than in the healthy postnatal kidneys, whereas the number of proliferating PECs is lower (15%) (Figure 2H).

### 2.3. Expression of Nestin in Human Podocytes and PECs of Developing, Postnatal, and CNF and FSGS Kidneys

**Figure 3:**
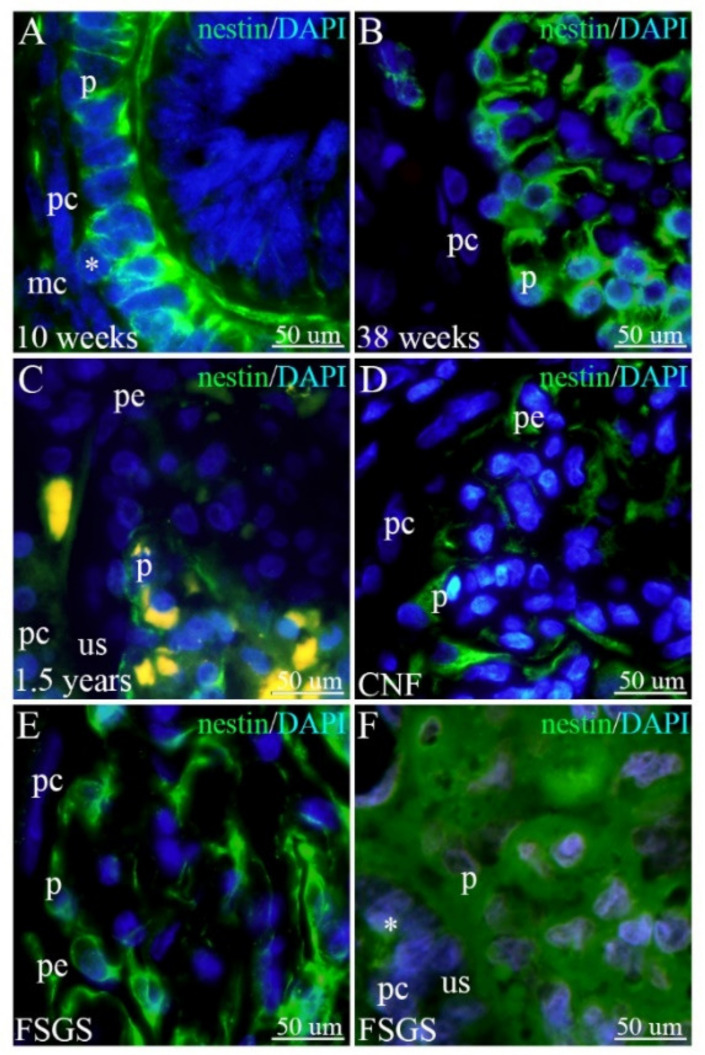
Expression of nestin in podocytes and PECs of developing and healthy postnatal kidneys and in kidneys affected by CNF and FSGS; mesenchymal cells (mc), podocytes (p), PECs (pc), peripolar cells (pe), urinary space (us). (**A**) 10th developmental week; the body of a podocyte bridges the thin urinary space (asterisk). (**B**) 38th developmental week; PECs become nestin-negative. (**C**) Postnatal healthy kidneys; expression of nestin decreases in the podocytes. (**D**) CNF kidneys; strong expression of nestin is present in the podocytes. (**E**) Early-stage FSGS kidneys; strong expression of nestin characterizes podocytes. (**F**) Late-stage FSGS kidneys; numerous nestin-positive cells bridge (asterisk) the urinary space.

In the 10th developmental week, the immature podocytes of the S-shaped body have the form of tall, closely packed epithelial cells, whereas PECs are presented as a simple squamous epithelium. Although podocytes show strong cytoplasmic expression of nestin, small cytoplasmic dots of nestin expression characterize the thin cytoplasm of PECs. The developing urinary space between the podocytes and PECs is bridged by the whole podocyte body, containing strong cytoplasmic nestin expression (podocytes at these early developmental stages mostly do not have developed cytoplasmic processes). Moderate nestin expression is also observed in the nearby mesenchymal cells (Figure 3A).

During further development, strong cytoplasmic nestin expression characterizes podocytes and also other glomerular cells (mesangial cells and endothelial cells of the glomerular capillaries), whereas PECs show no further nestin expression. Occasionally, nestin-positive cells can be observed in the surrounding mesenchyme, probably belonging to the cells in the vascular wall (Figure 3B).

In the postnatal period, podocytes display a significant reduction in the intensity of nestin expression, whereas PECs are completely devoid of nestin expression (Figure 3C).

In kidneys affected by CNF, strong nestin expression reappears in the podocytes and other glomerular cell populations. Strong nestin expression also characterizes cells in the transition zone between the podocytes and PECs (peripolar cells), whereas PECs are nestin-negative (Figure 3D).

In the early stages of FSGS, strong nestin expression is seen in the podocytes and cells bridging the urinary space (Figure 3E), whereas, along with the progression of FSGS, a blurred appearance of nestin expression is observed in the glomerular cells (including podocytes). Numerous cells bridge the space between the podocytes and PECs (Figure 3F).

### 2.4. Expression of Notch2 in the Developing and Postnatal Podocytes and Kidneys Affected by CNF and FSGS

**Figure 4:**
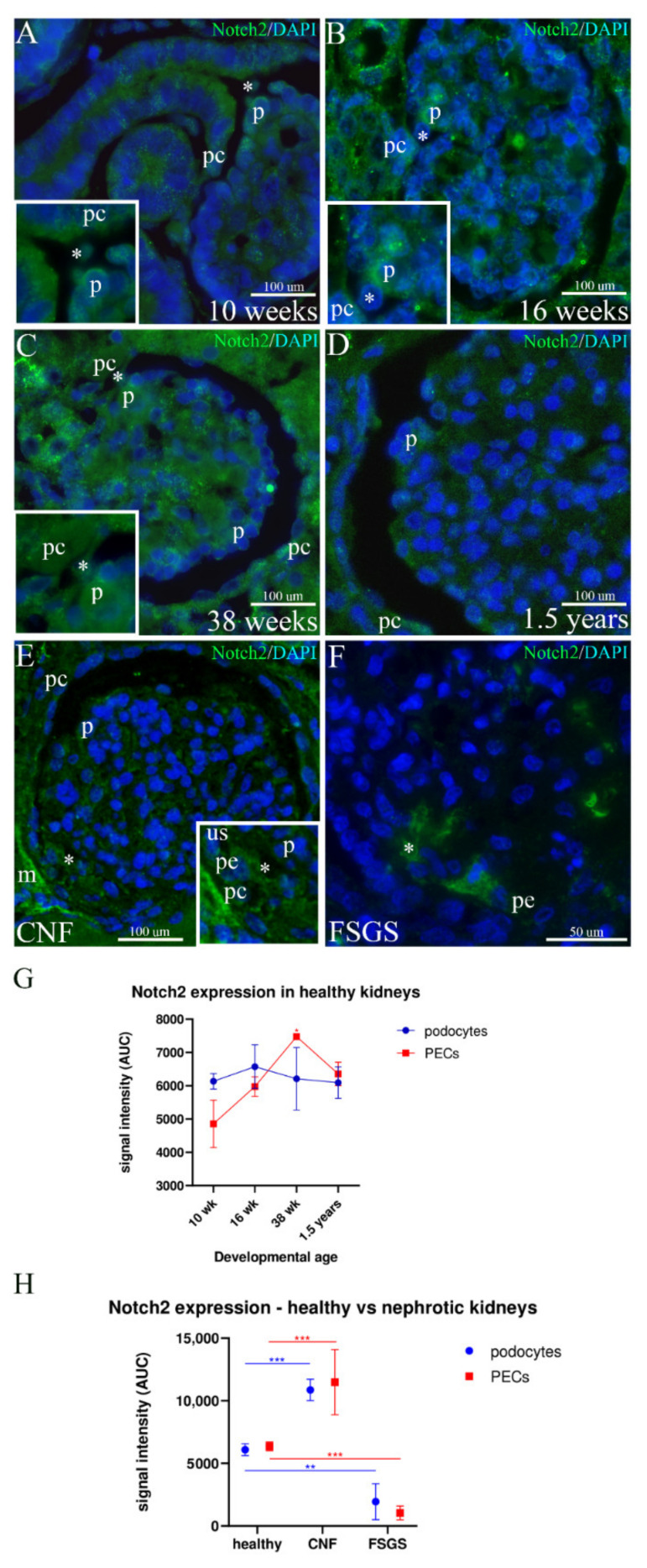
Expression of Notch2 in the developing podocytes and PECs in postnatal healthy kidneys and in kidneys affected by CNF and FSGS; mesenchyme (m), podocytes (p), PECs (pc), peripolar cells (pe), urinary space (us). (**A**) 10th developmental week; cells bridging the urinary space are seen (inset, asterisk). (**B**) 16th developmental week; cellular bridges (asterisk) connect podocytes and PECs (inset). (**C**) 38th developmental week; cellular processes (asterisk) span the urinary space (inset). (**D**) Postnatal healthy kidneys; moderate Notch2 expression is seen in podocytes and PECs. (**E**) CNF kidneys; peripolar cells (asterisk, inset) bridging the urinary space show moderate Notch2 expression. (**F**) FSGS kidneys; strong expression of Notch2 (asterisk) is seen in some glomerular cells, and only occasionally in the peripolar cells. (**G**) Overall expression pattern of Notch2 in healthy developing kidneys. (**H**) The comparison of the expression pattern between healthy and nephrotic kidneys shows significant differences in both podocyte and PEC subpopulations. Error bars show SD. * *p* < 0.05; ** *p* < 0.01; *** *p* < 0.001.

In the 10th week of developing kidneys, Notch2 expression characterizes several kidney structures including different parts of the nephron and collecting system. In the immature glomeruli, strong expression of Notch2 is seen only in some podocytes, whereas the expression in other podocytes and glomerular cells is weak or completely missing. Moderate expression of Notch2 is observed in PECs (Figure 4A).

In the mid-gestation period (16th developmental week), Notch2 expression increases in the whole glomerulus but is particularly strong in some podocytes (close to the vascular pole).

Notch2 expression is also seen in PECs and the cytoplasmic processes bridging the urinary space (Figure 4B).

By the end of the 38th week, unequal moderate Notch2 expression is observed in podocytes, whereas it increases in most of the PECs. Stronger Notch2 expression characterizes proximal tubules and distal tubules at the vascular pole of the glomerulus (Figure 4C).

In healthy postnatal kidneys, a reduction of Notch2 expression is seen in the glomerulus, with the exception of some individual podocytes that show moderate Notch2 reactivity. Moderate Notch2 expression is also observed in the PECs (Figure 4D).

In kidneys affected by CNF, moderate-to-strong expression in the form of fine granules characterizes all glomerular cells (including podocytes) and PECs. Strong expression of Notch2 is seen in the mesenchyme closely surrounding the affected glomerulus as well as some cells bridging the urinary space (Figure 4E).

In FSGS kidneys, the Notch2 signal is missing both in the podocytes and PECs, whereas it is strong in the cells participating in the formation of thick bridges between the podocytes and PECs corresponding to peripolar cells (pe) (Figure 4F).

Statistical analysis of Notch2 expression during development shows its gradual increase in PECs, having its peak in the 38th developmental week (*p* = 0.002) and then decreasing in the postnatal period. During the same period, Notch2 expression of nearly equal intensity is found in the podocytes, during both development and in the postnatal period (Figure 4G). Compared to the healthy postnatal kidneys, expression of Notch2 is significantly higher in both PECs (*p* < 0.001) and podocytes (*p* < 0.001) of CNF kidneys, whereas it is much lower in FSGS kidneys in both cell populations (*p* < 0.001 for PECs; *p* = 0.002 for podocytes) (Figure 4H).

### 2.5. WNT4 Expression in the Podocytes and PECs of Developing and Healthy Postnatal Kidneys and in Kidneys Affected by CNF and FSGS

**Figure 5:**
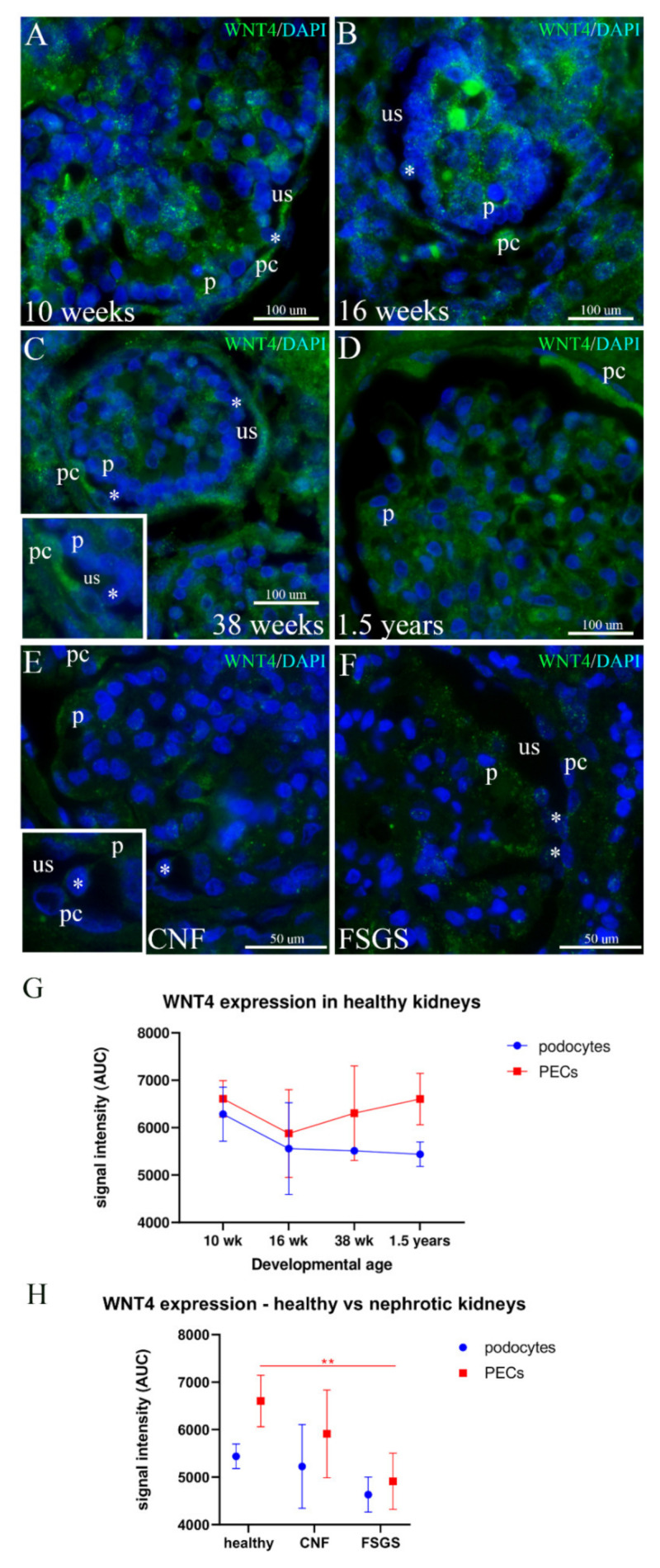
Expression of WNT4 in the developing podocytes and PECs in healthy postnatal kidneys and in CNF and FSGS kidneys; podocytes (p), PECs (pc), urinary space (us). (**A**) 10th developmental week; some podocytes are bridging (asterisk) the developing urinary space. (**B**) 16th developmental week; some podocytes bulge (asterisk) into the urinary space towards PECs. (**C**) 38th developmental week; at several places, podocytes bridge (asterisk) the urinary space. Wider contact between podocytes and parietal cells can be seen in some places (inset). (**D**) Postnatal healthy kidneys; podocyte expression of WNT4 is mild-to-moderate, whereas some PECs retain strong expression. (**E**) CNF kidneys; some cells (asterisk) are bridging the urinary space (inset). (**F**) FSGS kidneys; several cells and their cytoplasmic extensions bridge (asterisks) the urinary space. (**G**) Overall expression pattern of WNT4 in healthy developing kidneys. (**H**) Comparison of the expression pattern between healthy and nephrotic kidneys shows significant differences between PECs of healthy and CNF and FSGS kidneys. Error bars show SD. ** *p* < 0.01.

In the 10th week of human kidneys, WNT4 is strongly expressed in the nephrons and glomeruli as well as in the surrounding mesenchyme. Fine granular WNT4 expression is seen throughout the glomerulus. Some podocytes show more extensive expression than others, whereas all PECs show strong expression (Figure 5A).

In the 16th week of kidney development, WNT4 expression increases to very strong in some centrally positioned glomerular cells and some PECs, whereas the podocytes and most of the remaining PECs show moderate expression in the form of small dots (Figure 5B).

By the 38th week, more PECs acquire strong positivity, whereas podocytes display moderate expression (Figure 5C).

In healthy postnatal kidneys, most of the glomerular cells retain moderate WNT4 expression, with the exception of some centrally positioned cells, which show strong WNT4 expression. All PECs show strong WNT4 expression (Figure 5D).

In the CNF kidneys, the WNT4 signal is moderate-to-strong in some podocytes and PECs as well as in the cells bridging the urinary space (Figure 5E).

In kidneys affected by FSGS, mild-to-moderate granular WNT4 expression characterizes both the glomerular cell population and PECs (Figure 5F).

Statistically, in the earliest developmental stages, both podocytes and PECs show quite a strong expression of WNT4, which decreases during the first half of fetal development. However, by the end of the fetal period, podocytes continuously show low WNT4 expression, whereas PECs’ WNT4 expression reappears to reach an expression that was characteristic of early development. WNT4 expression is significantly higher in PECs (*p* = 0.049) compared to the podocytes of postnatal kidneys (Figure 5G). Strong expression of WNT4 in the PECs and its low expression in podocytes characterize healthy postnatal kidneys, whereas the expression decreases in CNF kidneys and particularly in PECs of FSGS kidneys (*p* = 0.007) (Figure 5H).

### 2.6. Snail Expression in the Developing and Healthy Postnatal Human Kidneys and Kidneys Affected by CNF and FSGS

**Figure 6:**
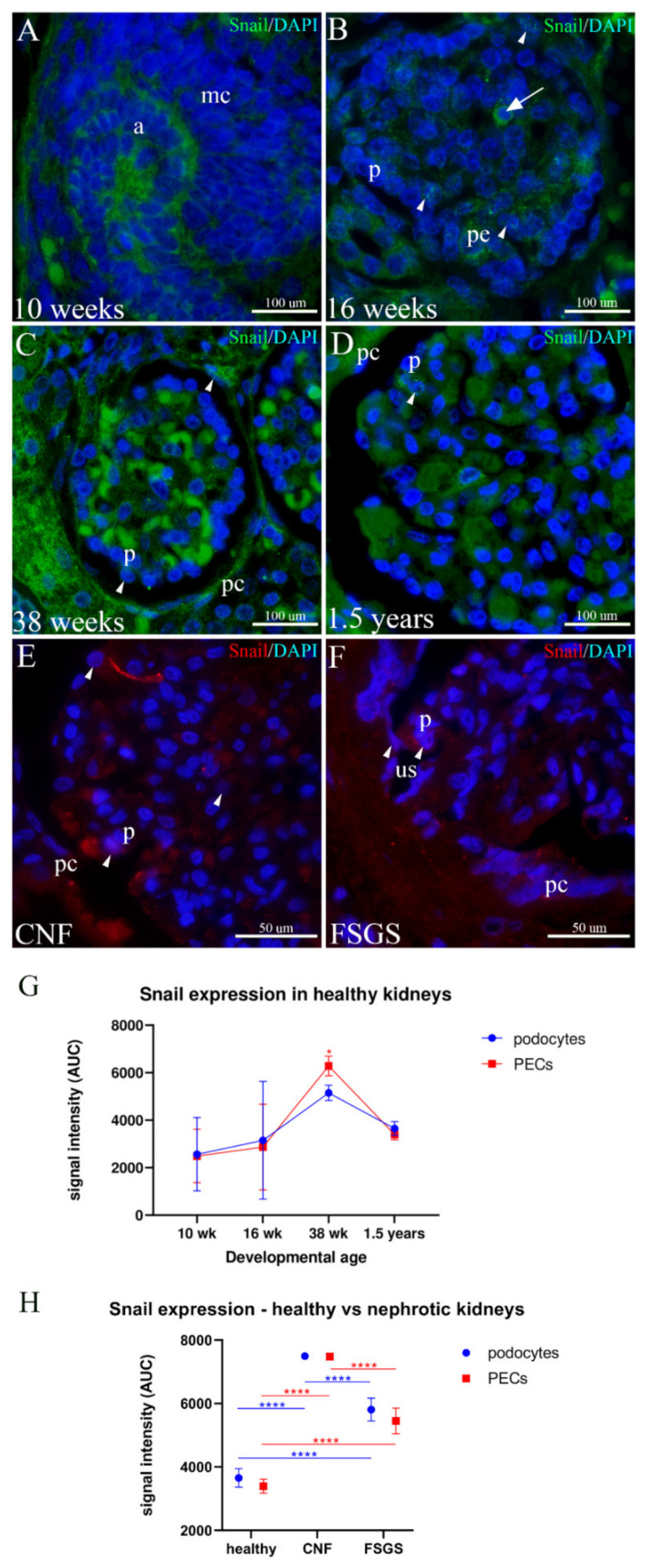
Expression of Snail in the developing podocytes and PECs in healthy postnatal and CNF and FSGS kidneys; metanephric cup (mc), ampulla (a), podocytes (p), PECs (pc), peripolar cells (pe), urinary space (us). (**A**) 10th developmental week; Snail expression is seen in the metanephric cup and ampulla of the collecting system. (**B**) 16th developmental week; moderate cytoplasmatic expression of Snail characterizes some centrally positioned glomerular cells (arrow), whereas some PECs and peripolar cells show nuclear Snail expression (arrowheads). (**C**) 38th developmental week; nuclear expression of Snail can be observed in some podocytes and PECs (arrowheads). (**D**) Postnatal healthy kidneys; weak nuclear Snail expression is present in some glomerular cells (arrowhead). (**E**) CNF kidneys; nuclear Snail expression can be found in some podocytes and glomerular cells (arrowheads). (**F**) FSGS kidneys; nuclear expression of Snail is observed in some podocytes and PECs that form bridges over the urinary space (arrowheads). (**G**) Overall expression pattern of Snail in the podocytes and PECs of healthy developing kidneys. (**H**) Comparison in the expression pattern between healthy and CNF and FSGS kidneys shows significant differences in both cell types. Error bars show SD. * *p* < 0.05; **** *p* < 0.0001.

In the cortex of the 10th week of developing human kidneys, the mesenchyme of the metanephric cup shows moderate-to-strong cytoplasmatic Snail expression as well as the ampulla at the tips of the collecting system (Figure 6A).

In the 16th week of human kidney development, Snail expression is weak in immature glomeruli and nephron tubules. Occasionally, some centrally positioned glomerular cells and cells in the peripolar region show strong cytoplasmatic Snail expression, whereas moderate nuclear expression is present in some glomerular cells and PECs (Figure 6B).

During further development, Snail expression remains weak both in the glomeruli and tubules. Compared to podocytes, PECs show moderate-to-strong expression of Snail, both cytoplasmatic and nuclear (Figure 6C).

In healthy postnatal kidneys, Snail expression is weak. Occasionally, rare podocytes and PECs, as well as some centrally positioned glomerular cells, show moderate Snail cytoplasmatic expression or weak nuclear expression (Figure 6D).

In kidneys affected by CNF, cytoplasmatic Snail expression increases to strong and nuclear expression to moderate in some podocytes and PECs as well as in some central glomerular cells (Figure 6E).

In FSGS kidneys, both cytoplasmatic and nuclear Snail expression increases to moderate in some podocytes and PECs, particularly in groups of cells bridging the urinary space between the two cell populations (Figure 6F).

Statistical measurements show that during human kidney development, Snail expression increases both in PECs and podocytes to reach maximal levels in the 38th week (*p* = 0.024). In the postnatal healthy period, Snail expression reduces again in both PECs and podocytes (Figure 6G). Compared to healthy kidneys, CNF kidneys show significantly stronger expression in both cell populations (*p* < 0.001). In kidneys affected by FSGF, the expression of Snail is significantly stronger than in healthy postnatal kidneys (*p* < 0.001) but is less extensive than in CNF (*p* < 0.001) (Figure 6H).

### 2.7. Expression of Nephrin and Synaptopodin Markers and Their Co-Expression with WNT4, Notch2, and Snail in Developing, Healthy Postnatal, and Human Kidneys Affected by CNF and FSGS

**Figure 7:**
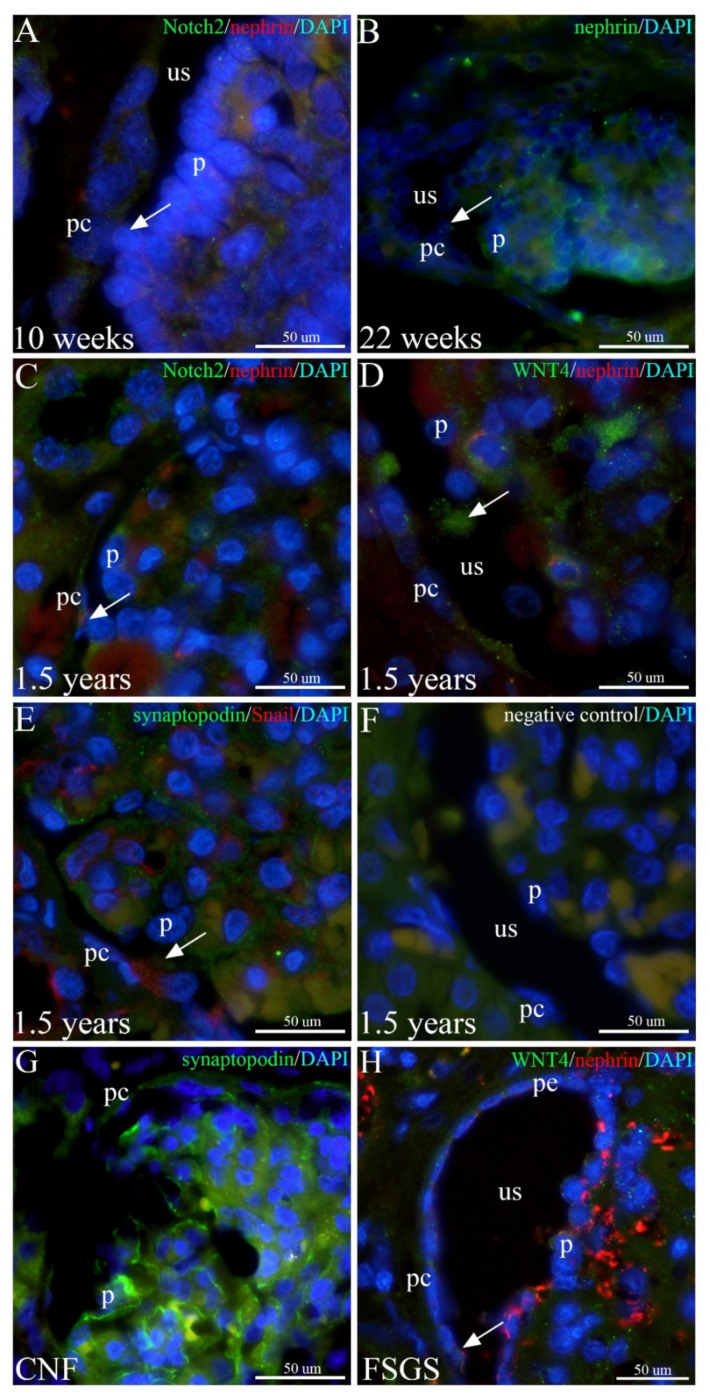
Expression of nephrin and synaptopodin and their co-expression with Notch2, WNT4, and Snail in developing, healthy postnatal human kidneys and kidneys affected by CNF and FSGS; podocytes (p), PECs (pc), peripolar cells (pe), urinary space (us). (**A**) 10th developmental week; Notch2 and nephrin co-localize in a cell (arrow) bridging the urinary space. (**B**) 22nd developmental week; nephrin expression characterizes the cytoplasm of a cell (arrow) bridging the urinary space. (**C**) Postnatal healthy kidneys; co-expression of Notch2 and nephrin is observed in the cytoplasm of the cell (arrow) connecting the glomerulus with PECs. (**D**) Postnatal healthy kidneys; strong expression of WNT4 is present in a cell bridging (arrow) the urinary space. (**E**) Postnatal healthy kidneys; co-localization of synaptopodin and Snail characterizes the cytoplasm of cells bridging (arrow) the urinary space. (**F**) Postnatal healthy kidneys; control staining characterized by omitting the primary antibody shows an absence of positive signals. (**G**) CNF kidneys; strong synaptopodin expression is visible in some podocytes. (**H**) FSGS kidneys; nephrin expression is present in some PECs (arrow).

Co-localization of mild Notch2 and nephrin expression characterizes podocytes on the surface of the glomerular tuft and cells spanning the urinary space towards the differentiating PECs in the 10th week of human kidney development (Figure 7A).

In the 22nd week of kidney development, nephrin expression characterizes podocytes and individual cells spanning the urinary space but not the PECs (Figure 7B).

In postnatal kidneys, stronger co-expression of nephrin and Notch2 is observed in podocytes and individual cells bridging the urinary space and coming into contact with PECs (Figure 7C).

A strong WNT4 signal is observed in cells bridging the urinary space and connecting the podocytes with PECs (Figure 7D) in healthy postnatal kidneys.

In the 1.5-year-old healthy kidney, co-expression of synaptopodin and Snail is observed in podocytes and a cell that bridges the urinary space and widely attaches to the PECs, which are synaptopodin-negative (Figure 7E).

Control staining of a 1.5-year-old normal kidney tissue sample shows an absence of cytoplasmic staining after staining with the secondary antibodies but without the application of the primary antibody. Orange staining of autofluorescent erythrocytes is also visible, similar to the other micrographs (Figure 7F).

In CNF, synaptopodin expression is strong in places, whereas it is missing in podocytes. Weaker synaptopodin expression characterizes the peripolar cells, whereas it gradually disappears in PECs along the length of Bowman’s capsule (Figure 7G).

In FSGS, a weak WNT4 signal co-expresses with atypically distributed nephrin strongly in the podocytes and mildly in the peripolar cells, whereas nephrin is missing in PECs (Figure 7H).

## 3. Discussion

The earliest stages of nephrogenesis are characterized by MET of cells that give rise to future nephrons. The proximal parts of these developing nephrons are the common origin of both podocytes and PECs. The precursors of these two cell populations can first be distinguished at the S-shaped body stage when they begin expressing different proteins, as the podocyte progenitors initiate a process of EMT [5]. It is believed that podocyte progenitor cells can be recruited from the stem cell population among PECs near the urinary pole, which migrate and gradually acquire the podocyte phenotype [9], thus becoming so-called “peripolar cells” or “parietal podocytes” [12,48]. In our study, we observed several bridges between the podocytes and PECs spanning the glomerular urinary space in developing and postnatal kidneys. In addition, in CNF and FSGS samples, the cellular bridges showed strong expression of Notch2, WNT4, and Snail, similar to their levels during normal development. Similar findings have been shown in vivo in animal models of glomerular disease [23]. In the cellular bridges, the expression of podocyte markers was also observed and it was suggested that those cells might serve as a replacement for the lost podocytes [22]. In murine models of crescentic glomerulonephritis, podocyte “bridges” formed first and partially displaced PECs, thus causing the formation of crescents [20]. The formation of cellular bridges has already been described during the normal development of the neural tube [49], suggesting the importance of this type of cellular communication for the normal development of different organs.

Our study showed that during the earliest stages of kidney development, the intense proliferation of both podocytes and PECs gradually decreased during further development and was accompanied by the increase in size and maturity of the glomeruli. The PEC proliferation was significantly higher compared to podocytes in all developmental stages, which corresponds to the fact that mature podocytes are postmitotic cells incapable of proper division [9]. In the nephrotic kidneys, the proliferation of podocytes was only slightly increased. This could represent an attempt to restore normal glomerular function by replacing defective podocytes. However, this process can cause more harmful than positive effects, since mature podocyte mitosis is inefficient and produces aneuploid cells that quickly detach from the glomerular surface and die [7].

The pattern of nestin expression in the observed glomerular subpopulations showed only weak expression in PECs in the earliest developmental stages, whereas they were completely devoid of nestin expression during further development and postnatally. This is in accordance with nestin being an immature cell marker that is replaced by other intermediate filaments with the progression of cell differentiation [24]. As for podocytes, nestin expression was present throughout the development and in the postnatal kidneys. Although the expression was strong during development, it became mild in the postnatal kidneys indicating the final differentiation of mature podocytes. It is unusual for fully differentiated cells to express nestin. However, among glomerular cell populations, this is the case only with podocytes [17]. A possible reason for such podocyte characteristics could be that they are the only glomerular cell type to undergo a special type of EMT [6]. It was shown that nestin has an important role in the structural integrity of podocytes and is able to increase their mechanical stability in rats [50]. In the podocytes of CNF and FSGS kidneys, nestin expression became strong again, similar to its expression during development. The blurred appearance of nestin expression in later stages of FSGS might be the result of the advanced sclerotic changes and accumulation of the extracellular matrix. The dedifferentiation process of human podocytes in CNF could explain the reappearance of nestin’s embryological expression pattern in pathologically changed kidneys [26].

WNT4 is one of the main signaling molecules that initiate nephrogenesis and MET, processes that enable the formation of renal vesicles. It also modulates cell adhesion molecules important for tubule formation and nephron maturation [51]. In mice, it is highly expressed in all the nephron structures during early nephrogenesis, but its expression significantly decreases as the nephrons mature and finally becomes undetectable in adults [52]. Our results on human developing kidneys showed a high expression of WNT4 during early nephrogenesis (8th to 10th week) in both podocytes and PECs, and a decrease in expression as nephrons undergo maturation (16th week), which is in line with the aforementioned studies. Although the podocyte expression of WNT4 continued to steadily decrease towards the postnatal stages, PEC expression increased to the levels observed during early nephrogenesis. It was suggested that in the absence of canonical Wnt signaling, prospective PEC differentiation is prevented and a switch towards podocyte fate is initiated [53]. Furthermore, it was shown that under pathological conditions, PECs could dedifferentiate and begin expressing some mesenchymal markers, which was accompanied by a significant decrease in WNT4 levels [54]. Therefore, we presume that high WNT4 levels in PECs serve to maintain the cell population in a stable differentiated state and prevent switching to a podocyte-specific fate. In CNF and FSGS kidneys, we found a slight but non-significant decrease in podocyte WNT4 levels but a significant decrease in PEC WNT4 levels. We propose that the decrease in WNT4 in PECs could be the result of their endeavor to replace the damaged podocytes by generating new ones from stem cells (present among PECs), which requires the inhibition of Wnt signaling [54]. Studies on the role of canonical Wnt signaling in nephrotic diseases have yielded opposing results. For example, in diabetic kidney disease mouse models, increased canonical Wnt signaling directly caused podocyte damage [55]. It was also suggested that sustaining Wnt signaling protects cells from high glucose-induced stress [56]. A study on WNT4 expression in human kidney diseases demonstrated that changes in WNT4 mRNA levels correlated with some clinical parameters depending on the specific disease [40]. Specifically, WNT4 levels in FSGS samples were found to have a negative correlation with albuminuria, meaning an increase in WNT4 could be protective.

Notch2 signaling also has a significant role in early nephrogenesis. It can probably mediate MET by WNT4-independent mechanisms [57] and it is necessary for the determination of the proximal cell’s fate during nephron segmentation [29]. Although its expression seems to be pivotal for the specification of podocytes during early nephrogenesis, the process of podocyte differentiation depends on its downregulation [58]. Upon completion of the glomerular development, Notch2 signaling decreases significantly in podocytes but increases in many patients and models of glomerular disease [59]. In our study, during the early stages of kidney development, Notch2 was widely expressed in several structures and its expression in podocytes was higher than in PECs. During later development (38th week), Notch2 expression decreased in podocytes and increased in PECs. In the healthy postnatal kidneys, Notch2 expression decreased in both cell populations, except in some individual podocytes. Similar expression patterns were also observed in other studies [29]. In our study, kidneys affected by CNF showed a significant increase in Notch2 expression, whereas a significant decrease characterized podocytes and PECs of FSGS kidneys. Similar to our study, multiple studies have also demonstrated increased Notch2 expression in proteinuric nephropathies [29,59,60]. The decrease in Notch2 expression in our FSGS samples correlated with the podocyte apoptosis described in the study where a loss of Notch2 signaling was associated with FSGS [61]. Interestingly, another study demonstrated that sustained Notch2 signaling could also lead to the apoptosis of podocytes [62]. Taking all of this into consideration, it is suggested that Notch2 signaling must be delicately regulated for normal podocyte development and function.

Snail is a transcription factor involved in both normal and pathological EMT. It is normally expressed in the metanephric mesenchyme prior to MET, keeping it in an undifferentiated state. In mice, the Snail becomes downregulated at the initiation of renal vesicle formation and remains silent during further development as well as in mature kidneys [45]. In contrast to these findings, our results showed a significant increase in Snail expression during later development (38th week), particularly in PECs. However, a study on mouse podocytes revealed that Slug, a member of the Snail family with equivalent functions, is normally upregulated in differentiated podocytes [63]. We speculate that the podocyte precursors need to express Snail in order to initiate EMT but subsequently lower its expression to allow podocyte maturation and expression of podocyte-specific proteins. This could explain Snail expression among PECs. However, more detailed studies on Snail and Slug expression in human developing kidneys are necessary to draw any meaningful conclusions. In CNF and FSGS kidneys, Snail expression was significantly upregulated in both podocytes and PECs, which corresponds to studies conducted on nephrotic rats and mice [46,47]. Snail expression in mature podocytes causes the reactivation of EMT and the dedifferentiation and downregulation of nephrin expression [47]. It is worth noting that CNF is caused by mutations in the NPHS1 (nephrin) gene, so podocytes of those kidneys do not have functional nephrin regardless of Snail expression. Interestingly, even though Snail is a transcription factor, we have observed significant cytoplasmatic staining in addition to typical nuclear staining. Cytoplasmatic staining of transcription factors, such as MSX homeodomain proteins, has previously been described [64]. In fact, Snail itself has been described as sometimes having a cytoplasmatic rather than nuclear localization [65], and this could be attributed to the different posttranslational modifications of Snail that can either induce its import into the nucleus or export into the cytoplasm [66]. Although cytoplasmatic Snail does not induce EMT [65], it may have other functions as it has been shown that Snail can directly act on other proteins, such as p53, by blocking their DNA-binding domain and inhibiting their function [67].

## 4. Materials and Methods

### 4.1. Human Tissue Processing

In our study, a total of 20 human kidney samples were investigated, including embryonic and fetal tissue, postnatal healthy, and pathologically altered tissue in CNF and FSGS (Table 2). Samples of human kidney tissues belong to the archive collection of human embryos and fetuses in the Department of Anatomy, Histology, and Embryology, at the School of Medicine, University of Split. Samples were collected with the permission of the Ethical and Drug Committee of the University Hospital in Split in accordance with the Helsinki Declaration (class: 003-08/16-03/0001, approval number: 2181-198-03-04-16-0024). Previously, embryonic and fetal tissue specimens were obtained after spontaneous abortions or tubal pregnancies, and only conceptuses with no signs of macerations, abnormalities, or morphological changes were included in the study. The ages of conceptuses between the 8th and 38th weeks were estimated by external measurements including crown–rump length and menstrual data. Postnatal kidney tissue was collected after the accidental death of a healthy 1.5-year-old child, as well as kidney tissue from patients with CNF and FSGS obtained after nephrectomy. All tissues were evaluated by a pathologist and the diagnosis of CNF was additionally confirmed by genetic analysis in a clinical laboratory, which revealed a pathogenic (according to ACMG classification) homozygous missense mutation c.1096 A > C; pSer366Arg in NPHS1 (the gene was detected in all three patients) [68].

### 4.2. Immunofluorescence Staining

Tissue samples were fixed in 4% paraformaldehyde in PBS (phosphate-buffered saline) and dehydrated at increasing ethanol concentrations. The dehydrated samples were embedded in paraffin and placed on slides after they were cut to a thickness of 5 µm. The immunofluorescence staining procedure started with deparaffinization in xylene and rehydration in water. Afterwards, samples were placed in a steamer in a sodium citrate buffer (pH 6.0) for 30 min. After washing in PBS, slides were coated in blocking buffer (ab64226, Abcam, Cambridge, UK) for a period of 30 min and then incubated overnight at room temperature in a humid chamber with the primary antibody or a combination of two primary antibodies, to analyze co-expression with double immunofluorescence staining (Table 3). The next day, the slides were washed in PBS and coated with appropriate secondary antibodies for 1 h. After washing again in PBS, the nuclei were stained with DAPI (4′6-diamidino-2-phenylindole), a DNA-binding solution. Next, the slides were rinsed with distilled water and covered with coverslips. Exclusion of primary antibodies from the staining procedure was used as a control for the specificity of staining. The slides were observed under a fluorescence microscope (Olympus BX61, Tokyo, Japan) and photographed using a DP71 digital camera (Nikon, Tokyo, Japan) with NIS-Elements F software [68].

### 4.3. Preparation of the Tissue for Electron Microscopy (TEM)

Tissues samples for electron microscopy were fixed for 24 h in 4% paraformaldehyde in PBS at 4 °C, washed with PBS, and then cut with a vibratome (Vibratome Series 1000, Pelco 101, Ted Pella, Inc., Redding, CA, USA) into semi-thin sections of 1 µm thickness and stained using toluidine blue. Next, the samples were fixed in 1% osmium tetroxide for 1 h, dehydrated in ethanol, and incorporated into LX 112 resin. Ultra-thin sections (0.05 µm) were sliced from semi-thin sections and stained with uranyl acetate and lead citrate. The samples were observed with a transmission electron microscope (TEM; JEM JEOL 1400, Japan) [68].

### 4.4. Data Acquisition and Quantitative Analysis

We analyzed the proliferation of glomerular subpopulations by calculating the percentage of proliferating cells. For each observed glomerulus, three independent researchers counted the number of all podocyte nuclei and podocyte nuclei that expressed Ki-67 using ImageJ software (NIH, Bethesda, MD, USA). The percentage of Ki-67-positive podocytes was then calculated as the ratio between the two numbers, and the overall percentage was expressed as the mean ± SD. The same procedure was performed for PECs. In order to analyze the expression of Notch2, WNT4, and Snail, figures had to be prepared beforehand. All figures were captured at x40 magnification and for each figure analyzed, we extracted three to five 50-pixel by 150-pixel rectangular areas containing podocytes and the same number of areas containing PECs, using Adobe Photoshop (version 9.0). We then acquired intensity histograms for the green or red fluorescence channels of the extracted podocytes and PECs in ImageJ software (NIH, Bethesda, MD, USA). Background thresholds were determined based on negative control images by three experienced histologists. The area under the curve (AUC) of the fluorescence intensity histograms was used to quantify protein expression. The AUC analysis routine in GraphPad Prism 9.3.1 software (Graph Pad, La Jolla, CA, USA) was used to calculate AUCs [69]. Afterwards, AUC values were divided by the number of rectangular areas used for each particular figure in order to allow the comparison between different groups of samples. We used three technical replicates per analyzed developmental and pathologic sample. Only podocytes at the periphery of glomeruli were analyzed. All graphs were made using GraphPad Prism 9.3.1 software (Graph Pad, La Jolla, CA, USA).

Additionally, we measured the length of the intercellular bridges in x40 magnification immunofluorescent and high-magnification TEM micrographs with ImageJ software (NIH, Bethesda, MD, USA). We only measured the bridges that spanned the entire urinary space and took the measurement as the shortest distance between the points of contact on the glomerular tuft and the wall of Bowman’s capsule.

Two-way ANOVA with Fisher’s LSD post hoc test was used to determine peaks or nadirs in protein expression of podocytes or PECs during normal kidney development, and to ascertain whether there were significant differences in protein expression between the observed groups of samples. Statistical significance was set at *p* < 0.05.

## 5. Conclusions

The proliferation rate and nestin expression among podocytes and PECs decrease during normal human kidney development. WNT4 levels continuously decrease in podocytes, whereas in PECs they increase back to their early developmental levels. Notch2 expression is initially higher in podocytes than in PECs but this relationship changes during later development and postnatally. Snail is continuously downregulated in both cell populations, with the exception of a transient increase during later fetal development (38th week). Numerous cell bridges and extensions of podocytes and PECs can be observed spanning the urinary space in the developing and postnatal glomeruli. In CNF and FSGS kidneys, the expression of all observed factors is changed to the levels present in the developing glomeruli, whereas increased formation characterizes cellular bridges. Our results suggest that the mechanisms present during normal development are reactivated under pathological conditions in an attempt to restore glomerular integrity.

## Figures and Tables

**Table 1:** Existing information about the analyzed proteins.

Protein	Type of Protein	Functions	Expression in Developing Kidneys (Animal Models)	Expression in Nephrotic Kidneys (Human Samples and Animal Models)
nestin	intermediate filament	expressed in stem and precursor cells as a placeholder for more mature intermediated filaments	at early stages expressed both in podocytes and PECs; at later stages only in podocytes	increased expression in podocytes
Notch2	transmembrane receptor	mediates proliferation, apoptosis, EMT, and differentiation	highly expressed in nephrons at earlier stages, decreases as nephrons mature	increased expression in both podocytes and PECs
WNT4	paracrine signaling molecule	mediates axis prolongation, organogenesis, MET, proliferation	highly expressed in nephrons at earlier stages, decreases as nephrons mature	both increases and decreases have been described, depending on the specific disease
Snail	transcription factor	mediates formation of mesoderm and neural crest, EMT, cell survival and division	present in metanephric mesenchyme before induction of MET, downregulated as nephrons mature	increased expression in podocytes

**Table 2:** The human kidney samples analyzed in the study.

Weeks/Months/Years	Number of Kidney Samples	Status
8 weeks	4	Human conceptuses
10 weeks	3	
16 weeks	1	
22 weeks	2	
38 weeks	2	
1 year	3	Healthy postnatal kidneys
1 year 6 months		
7 years		
3 years	3	CNF kidneys
5 years		
1 year	4	FSGS kidneys
1 year 4 months		
6 years		
10 years		

**Table 3:** Primary and secondary antibodies used in the study.

	Antibodies	Host	Code no.	Dilution	Source
Primary	Anti-NOTCH2	Rabbit	ab8926	1:100	Abcam, Cambridge, UK
	Anti-WNT4	Rabbit	ab91226	1:25	Abcam, Cambridge, UK
	Anti-SNAIL	Goat	ab53519	1:400	Abcam, Cambridge, UK
	Anti-nestin	Rabbit	ab93157	1:200	Abcam, Cambridge, UK
	Anti-Ki-67	Mouse	M7240	1:100	DAKO, Santa Clara, CA, USA
	Anti-nephrin	Goat	sc-32530	1:200	Santa Cruz Biotechnology, Inc., Dallas, TX, USA
	Anti-synaptopodin	Rabbit	ab117702	1:300	Abcam, Cambridge, UK
Secondary	Alexa Fluor^®^488 AffiniPure Anti- Mouse lgG (H + L)	Donkey	715-545-150	1:400	Jackson Immuno Research Laboratories, Ely, UK
	Alexa Fluor^®^488 AffiniPure Anti- Goat lgG (H + L)	Donkey	705-545-003	1:400	Jackson Immuno Research Laboratories, Ely, UK
	Alexa Fluor^®^488 AffiniPure Anti- Rabbit lgG (H + L)	Donkey	711-545-152	1:400	Jackson Immuno Research Laboratories, Ely, UK
	Rhodamine Red™-X (RRX) AffiniPure Anti-Goat IgG (H + L)	Donkey	705-295-003	1:400	Jackson Immuno Research Laboratories, Ely, UK
	Rhodamine Red™-X (RRX) AffiniPure Anti-Mouse IgG (H + L)	Donkey	715-295-151	1:400	Jackson Immuno Research Laboratories, Ely, UK

## Data Availability

The data presented in this study are available on request from the corresponding author. The data are not publicly available due to ethical concerns.

## Supplementary Materials

The following supporting information can be downloaded at: https://www.mdpi.com/article/10.3390/ijms23147501/s1.
