# Supplementary material for: Differences in Immunohistochemical and Ultrastructural Features between Podocytes and Parietal Epithelial Cells (PECs) Are Observed in Developing, Healthy Postnatal, and Pathologically Changed Human Kidneys
Source: Int J Mol Sci. 2022 Jul 6;23(14):7501. doi: 10.3390/ijms23147501 (PMC9322852; doi:10.3390/ijms23147501)
Supplement: Supplementary file 1 [file ijms-23-07501-s001.zip › ijms-1742235-supplementary.pdf]

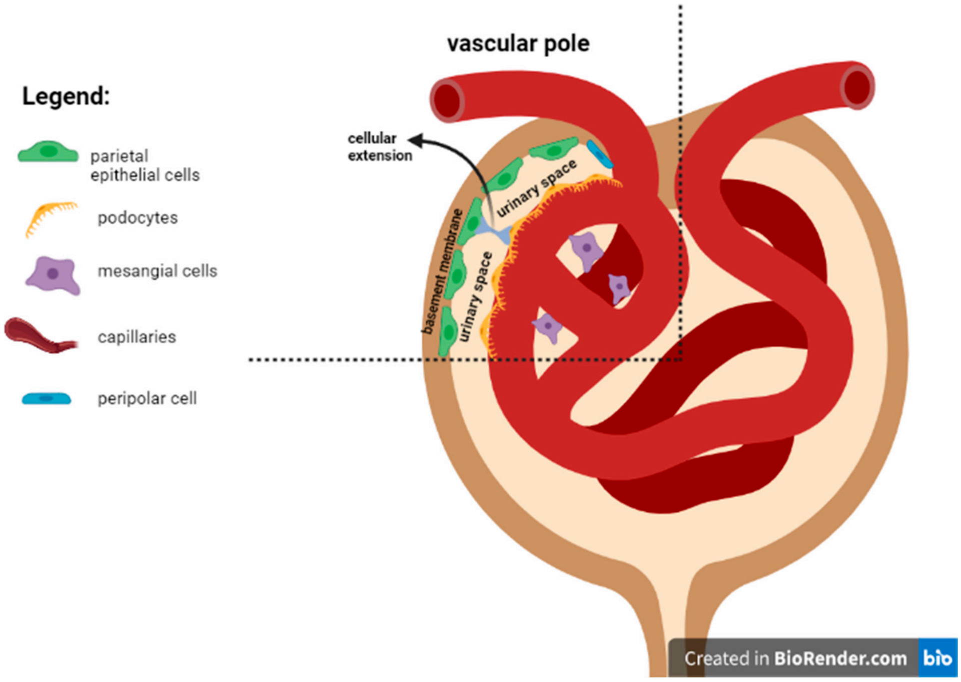

Figure S1. Schematic drawing of the glomerular structure.

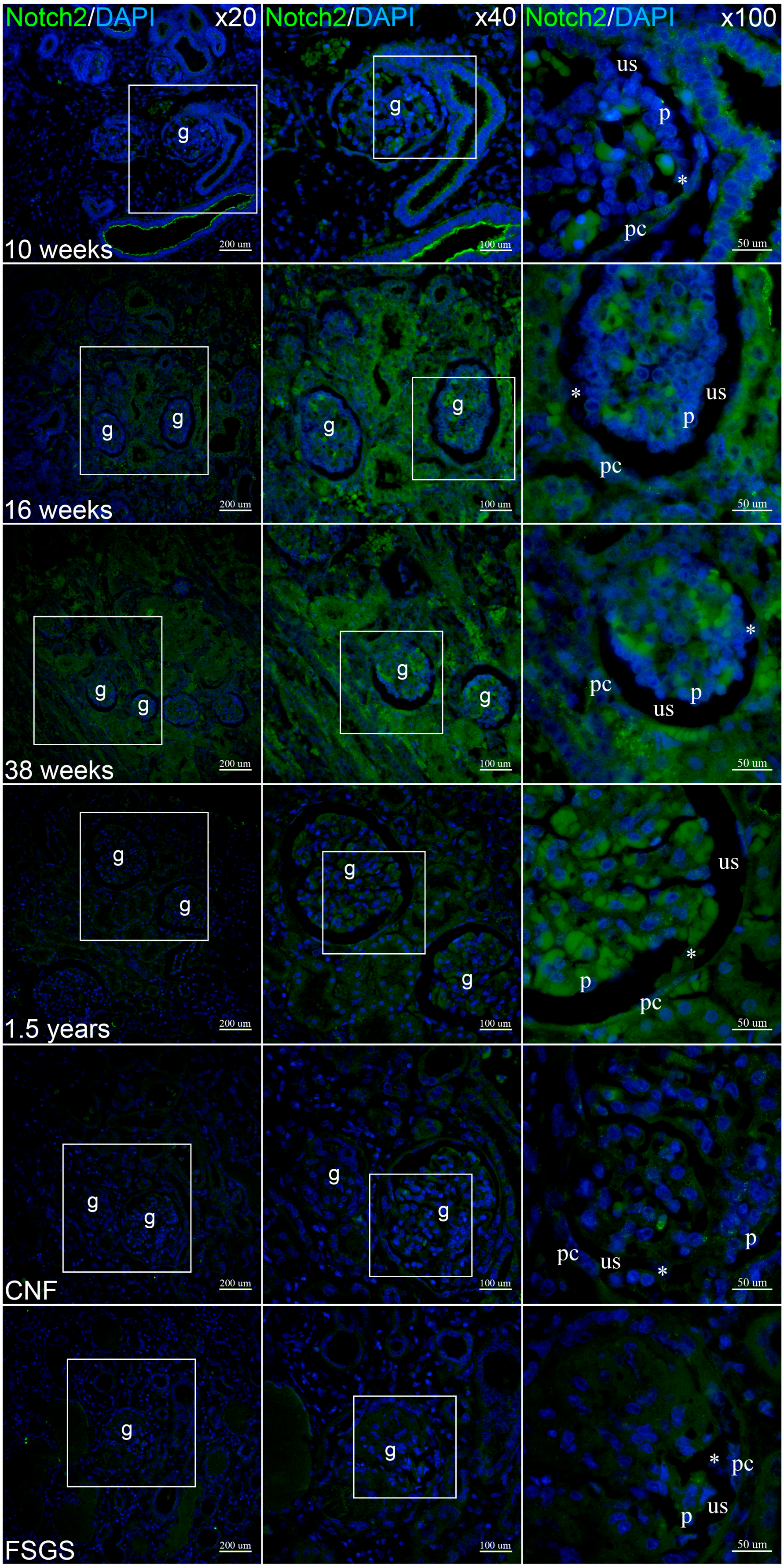

Figure S2. Lower and high magnification immunofluorescent images demonstrating kidney architecture of the observed samples.
